# Anthropometric Measures, Muscle Resistance, and Balance in Physically Active, Aged Adults

**DOI:** 10.3390/sports11060113

**Published:** 2023-06-06

**Authors:** Filipe Rodrigues, Raul Antunes, Rui Matos, Miguel Jacinto, Diogo Monteiro, Pedro Forte, António Miguel Monteiro, Tiago M. Barbosa

**Affiliations:** 1 ESECS, Polytechnic of Leiria, 2411-901 Leiria, Portugal; 2 Life Quality Research Center, 2040-413 Leiria, Portugal; 3 Center for Innovative Care and Health Technology, 2410-541 Leiria, Portugal; 4 Research Centre in Sports Sciences, Health, and Human Development, 6201-001 Covilhã, Portugal; 5 Department of Sport Sciences and Physical Education, Instituto Politécnico de Bragança, 5300-253 Bragança, Portugal; 6 ISCE Douro, 4560-708 Penafiel, Portugal

**Keywords:** muscle strength, balance, fall, elderly, health, aging

## Abstract

Objectives: This study aimed to examine the relationship between age, body mass index, muscle strength, and balance in physically active, aged adults. Methods: Eighty-five participants were recruited for this study, having an average age of 70.31 years (SD = 9.90), ranging from 50 to 92 years. Twenty-six (30.6%) participants were male and fifty-nine (69.4%) were female. The participants had an average body mass index of 27.30 kg/m^2^ (SD = 3.62), ranging from 20.32 to 38.58 kg/m^2^. Participants undertook the Timed-Up and Go to test balance, and the chair-stand test to assess lower body strength. Hierarchical regression analyses were conducted. Three models (Model 1, 2, and 3) were tested to assess their relationships with balance: M1—Lower body muscle strength; M2—Lower body muscle strength and body mass index; M3—Lower body muscle strength, body mass index, and age. Results: All hierarchical models displayed significant variance. The third model explained 50.9% of the variance in dynamic balance, [F(3, 81) = 27.94, *p* < 0.001, R = 0.71, R_a_^2^ = 0.51]. The difference in R_a_^2^ between the first, second, and third models was statistically significant (*p* < 0.05). Age, body mass index, and lower body muscle strength had significant (*p* < 0.05) correlations with balance. In terms of the significant impact of each predictor, age had the strongest association with balance (*p* < 0.05). Conclusions: The results are useful to understand mechanisms or diagnose people at risk of fall.

## 1. Introduction

The process of aging is an inherent phenomenon affecting all living organisms. Advancing age brings about a series of physiological changes that can significantly impact overall health and well-being. These alterations have the potential to affect multiple bodily systems, including the cardiovascular, musculoskeletal, nervous, and immune systems. In the context of the cardiovascular system, aging is associated with a reduction in heart size and function, elevated blood pressure, and thickening of blood vessel walls. Concurrently, musculoskeletal changes observed during the aging process encompass the loss of muscle mass and strength known as sarcopenia, as well as the decline in bone mineral density referred to as osteopenia. Such musculoskeletal changes contribute to an increased susceptibility to falls and fractures, thus further compromising overall health [1]. Age-related modifications in the nervous system entail cognitive impairments, including memory decline and diminished processing speed, alongside changes in brain structure and function. Collectively, these alterations can lead to reduced physical fitness and heightened vulnerability to diseases and disabilities [2], particularly among the elderly population [3]. Consequently, the physical fitness of older adults is significantly impacted [4,5].

Physical fitness in older adults encompasses the capacity to engage in routine physical activities, such as walking, climbing stairs, and carrying groceries, without discomfort or pain. This measure encompasses various factors, including strength, endurance, flexibility, balance, and coordination. Sustaining physical fitness represents a fundamental aspect of healthy aging and plays a pivotal role in promoting overall well-being [6]. Muscle strength emerges as a critical component of physical fitness, as it underpins balance, mobility, and overall physical performance [7]. The aging process is inherently associated with a decline in muscle mass and strength, culminating in reduced physical fitness and an augmented risk of falls. Moreover, evidence suggests a potential association between body mass index and diminished physical fitness, positing that increased fat mass may impose mobility limitations, subsequently inducing a decline in physical activity [8]. Nevertheless, the decrease in physical activity levels contributes to an increase in fat mass and a decline in muscle strength, further exacerbating the deterioration of physical fitness [9,10]. Balance assumes a crucial role in physical fitness and healthy aging. Throughout the aging process, both physical fitness and balance decline, augmenting the risk of falls, compromising independence, and diminishing overall quality of life. Research has consistently demonstrated a robust association between physical fitness and balance among older adults [7]. Specifically, individuals with superior physical fitness tend to exhibit better balance, whereas those with inferior physical fitness face an elevated risk of falls and injuries related to balance impairment.

Regular engagement in physical activity has been demonstrated to enhance muscular function and strength, both of which play a pivotal role in improving balance [11]. The existing literature has presented various exercise programs, including resistance, power, and aerobic training modalities, aimed at enhancing functional fitness among older adults [12], particularly in relation to balance and quality of life [13]. Furthermore, investigations have explored the associations between balance, strength, and body composition [14]. However, the literature engages with a variety of tests and methodologies for assessing balance, thereby impeding the ability to derive practical implications regarding the significance of physical activity. Both static and dynamic balance exhibit relationships with the risk of falls [14,15]. Common methods for assessing balance are based on static posture and dynamic activities [16]. The use of photogrammetry, although valid, is time-consuming and impractical for data collection in empirical settings [17]. A simple and valid method for evaluating walking capacity is the Timed-Up and Go (TUG) test [12,18,19,20]. The TUG test, which assesses both motor and cognitive functions, can be employed to evaluate dynamic balance due to its association with balance and falls. Moreover, it offers a cost-effective alternative to photogrammetry or other related balance measures.

In light of the aforementioned associations, there is a need to comprehend the extent of the relationships between lower limb strength, body mass index, and age concerning balance among older adults. Such understanding can facilitate physicians in effectively diagnosing balance impairments, which can significantly impact the quality of life of elderly individuals residing in the community. Nevertheless, the extent of these associations within the aged population remains unclear. It is crucial to elucidate the effects of lower limb strength, age, and body mass index on balance within a population vulnerable to falls due to age-related factors. The results can unveil patterns and relationships in the data, particularly the associations between physiological indicators that contribute to fall risks. This study aims to identify potential risk factors for falls and other balance-related injuries among physically active, older adults, thereby aiding in the development of effective fall prevention programs and interventions that can enhance overall physical fitness and quality of life within this population. Additionally, the findings can serve as a basis for generating hypotheses for further research regarding the implications of healthy aging on body mass index, muscle strength, and subsequently, balance.

An innovative aspect of this study lies in its focus on physically active, elderly individuals, a population that has received relatively limited attention in prior research [7,9]. By examining the experiences and behaviors of this specific group, new insights can be gained regarding the ways in which physical activity can benefit older adults and contribute to healthy aging. Furthermore, this study includes individuals aged 50 and older, a demographic often overlooked in studies on physical activity and healthy aging. By incorporating this age range, the study aims to provide a more comprehensive and nuanced understanding of the advantages of physical activity for older adults. The primary objective of this study is to investigate the relationships between age, body mass index, muscle strength, and balance among physically active, older adults. It is hypothesized that balance can be predicted by lower limb strength, age, and body mass index, with muscle strength demonstrating a positive and significant association with balance.

## 2. Materials and Methods

### 2.1. Participants

To ensure the validity and reliability of our study, we utilized the a priori sampling calculator for multiple hierarchical regression analysis [19] to determine the minimum required sample size. Informed by existing evidence [12], we considered the number of predictor variables in set A, the number of predictor variables in set B, probability level (α = 0.05), desired statistical power (1−β = 0.80), and effect size (small effect with a value of 0.15). The calculator indicated a minimum sample size of 68 participants for the study findings to be considered valid and reliable. Our study included 85 participants, with an average age of 70.31 years (SD = 9.90), ranging from 50 to 92 years. Among the participants, 30.6% (*n* = 26) were male and 69.4% (*n* = 59) were female. The average body mass index of the participants was 27.30 kg/m² (SD = 3.62), ranging from 20.32 to 38.58 kg/m². The participants were actively involved in physical activities, including exercising at the gym or fitness center, participating in community exercise programs, or following exercise routines at home. Physically active elderly individuals were defined as those engaging in regular physical activity and exercise at least twice a week.

In order to be eligible for participation in the study, individuals had to meet the following inclusion criteria: (a) be at least 50 years of age; (b) be in generally good health and free from major chronic illnesses that would impede their participation; (c) have the ability to walk independently or with assistance; (d) possess the ability to understand and follow instructions, as well as provide informed consent; (e) reside in their own homes rather than in a long-term care facility; (f) not be taking any medications that influence muscle mass or function; (g) be actively involved in physical programs designed to promote acceptable levels of physical fitness. To ensure safety, individuals with a history of chronic neuromuscular, cardiovascular, or metabolic conditions that could potentially pose a risk during the study or evaluation periods were excluded from participation. These carefully considered inclusion and exclusion criteria aimed to ensure the safety of participants and enhance the accuracy and reliability of the study findings.

Prior to conducting the investigation, ethical institutional approval (128/CES/INV/2013) was obtained. Given the researchers’ access to potential volunteers through exercise programs associated with higher education institutions, a convenience sampling strategy was employed to collect data. Objectives and data collection processes were provided to the principals and managers for their review and approval. Subsequently, potential participants were approached during classes and invited to voluntarily participate in the study. They were provided with detailed information regarding the study objectives and procedures and were required to provide individual informed consent before participation. No compensation was provided to participants for their involvement in the study.

### 2.2. Research Design

The present cross-sectional study was conducted with the aim of investigating the factors underlying dynamic balance in community-dwelling, older individuals. Specifically, we explored the association and dependence of dynamic balance on age, body mass index, and lower limb strength. To ensure consistency and reliability, all participants were evaluated by the same evaluator. On the day of testing, participants engaged in an 8 to 10-minute warm-up session under the supervision of a trained physical education teacher.

Previous research has examined balance in older populations using both static and dynamic assessment methods [4]. However, the literature has emphasized the significance of dynamic balance as it relates to independent engagement in daily activities and overall physical fitness levels [1]. Consequently, we selected the Timed-Up and Go test as the preferred method for assessing dynamic balance in this population. The functional fitness test for older individuals incorporates a battery of tests designed to evaluate various components of physical fitness, including lower limb strength [20]. Lower limb strength was measured using the chair-stand exercise, a resistance exercise commonly utilized in the literature for assessing lower limb strength [20].

### 2.3. Instruments and Procedures

Weight measurements were obtained from participants while wearing lightweight clothing and without shoes, using a digital bioimpedance scale (Tanita BC-50, Arlington Heights, IL, USA), prior to breakfast. Height measurements were taken using a digital scale with an attached stadiometer (SECA^®^, Chino, CA, USA) to determine the distance between the vertex and the reference plane on the ground. Body mass index (BMI) was calculated using the standard formula: BMI = weight (kg)/height^2^ (m). Age was recorded in years.

To assess lower body strength and functional mobility, the chair-stand test was selected [20]. This test involves determining the number of times an individual can stand up from a chair and sit back down within a 30-s period. The researcher provided clear instructions to the participant, demonstrating proper standing and sitting techniques from the chair. Participants were informed that the objective was to perform as many stand-ups and sit-downs as possible within the given time. They were instructed to sit on the chair with their back straight and feet flat on the floor. The participants were then instructed to stand up fully and sit back down completely, without using their arms to push off the chair. The stopwatch was initiated after verbal cues of “Ready, go!” Participants were required to continue standing up and sitting down as many times as possible within the 30-s duration. At the end of the allotted time, the researchers announced “Stop!” and recorded the total number of completed stand-ups and sit-downs. The test was repeated up to two times, with the highest score used for analysis. Previous studies have established the reliability and validity of the chair-stand test as a measure of lower body strength and functional mobility in older adults [21]. Additionally, it has been identified as a predictor of falls and functional decline, with a lower number of completed stands indicating a higher risk of such incidents [21]. Due to its simplicity, cost-effectiveness, and ease of administration, the chair-stand test is widely utilized in geriatric research and practice to assess lower body function.

Furthermore, the Timed-Up and Go (TUG) test was employed to evaluate balance [20]. The TUG test also serves as a measure of agility [22]. This test involves timing how long it takes an individual to stand up from a chair, walk a distance of 2.44 m, turn around, return to the chair, and sit back down. Participants were instructed to sit on the chair with proper posture, with their feet flat on the floor. The researcher explained that they would need to stand up from the chair, walk the specified distance, turn around, and then return to the chair to sit down again. The stopwatch was initiated after verbal cues of “Ready, go!” Participants were required to stand up, walk the designated distance, turn around, and walk back to the chair. They then needed to sit back down, with the stopwatch stopped when their back made contact with the chair’s backrest. Timing was recorded to the nearest tenth of a second. This test is considered a robust and reliable measure of balance in older individuals [21].

### 2.4. Statistical Analysis

To assess the normality of the distribution, the Kolmogorov-Smirnov test was employed. Bivariate Pearson correlations were conducted to examine the relationships among the selected variables of interest. The postulated relationships were tested using hierarchical multiple regression analyses. Prior to commencing the regression analysis, the tolerance test and variance inflation factor (VIF) values were examined to identify and address potential issues of multicollinearity [23]. Multicollinearity was considered absent when the tolerance values for the independent variables exceeded 0.1. Furthermore, the Durbin-Watson statistic test was performed to assess autocorrelation, with a range of 1.50 to 2.50 being deemed appropriate [24].

Hierarchical regression analysis is a statistical technique used to investigate the association between one or more independent variables and a dependent variable. In this analysis, the predictors are introduced into the model in a predetermined order. In the present study, three models were constructed, with each model incorporating an additional independent predictor variable. The first model included solely the first predictor variable, the second model included both the first and second predictor variables, and the third model encompassed all three predictor variables. The purpose of employing hierarchical regression analysis with one predictor in each model is to examine the unique contribution of each predictor to the predictive capacity of the model. By systematically adding predictors to the model, researchers can assess the impact of each variable on the relationship between the independent and dependent variables. The stepwise method was employed as it allows for the inclusion of variables based on theoretical assumptions. Balance was chosen as the dependent variable in the analysis. In Model 1, lower muscle strength was selected as the sole independent variable. Model 2 included both lower muscle strength and body mass index as independent variables. Finally, in Model 3, age was added to the previous two independent variables. The significance level for rejecting the null hypothesis was set at 5% for all tests.

## 3. Results

Table 1 presents the descriptive statistics and bivariate correlations among the variables. The normality of the distribution was confirmed as evidenced by the non-significant result of the Kolmogorov-Smirnov test (*p* > 0.05). Consistent with expectations, several significant bivariate correlations were observed. Age exhibited a positive association with body mass index and the Timed-Up and Go test, and a negative association with the chair-stand test (*p* < 0.001). Body mass index demonstrated a positive association with the Timed-Up and Go test and a negative association with the chair-stand test (*p* < 0.001). Furthermore, the chair-stand test showed a negative association with the Timed-Up and Go test (*p* < 0.001).

Table 2 displays the output of the hierarchical multiple regression analysis. Tolerance values ranged from 0.78 to 1.00. Furthermore, the VIF values ranged between 1.00 and 1.27. Hence, there were no multicollinearity issues in the dataset. The Durbin-Watson test result yielded 1.30, which is deemed satisfactory. We examined the significance of the model to see if it was substantially different from the null hypothesis. We looked at the *R*^2^ value to evaluate variation in dynamic balance.

The regression model in B1 was statistically significant, showing that lower body strength accounted for 13.6% of the variance in balance [F(1, 83) = 14.18, *p* < 0.001, R = 0.38, R_a_^2^ = 0.136]. The second model (B2) explained 32.1% of the variance in balance, [F(2, 82) = 19.39, *p* < 0.001, R = 0.57, R_a_^2^ = 0.32]. The difference in R_a_^2^ between the first and second models was statistically significant (*p* < 0.05). The third model (B3) explained 50.9% of the variance in balance [F(3, 81) = 27.94, *p* < 0.001, R = 0.71, R_a_^2^ = 0.51]. The difference in R_a_^2^ between the first, second, and third models was statistically significant (*p* < 0.05). As such, when compared to the previous models, the final one is the most parsimonious. In the three examined models, all independent variables had a significant (*p* < 0.05) effect on balance.

## 4. Discussion

The primary objective of this study was to investigate the associations between age, body mass index, muscle strength, and dynamic balance in older adults. Consistent with our hypotheses, increased age and BMI were found to have a positive and significant correlation with balance. Conversely, lower body muscle strength exhibited a negative and significant association with balance, indicating a reduced risk of falling among older adults. Notably, the participants in this study demonstrated better performance in terms of muscle strength and dynamic balance compared to those reported in previous studies [20,22,25]. This favorable outcome could be attributed to the participants’ engagement in regular exercise sessions aimed at promoting physical fitness. The beneficial effects of physical activity on maintaining satisfactory levels of physical fitness, even in older adults, have been well-documented [26,27]. Consequently, it is expected that physically active older adults would exhibit greater muscle strength and dynamic balance compared to their sedentary counterparts [26]. The ability of older adults to adjust their perceived effort within the limits of their support base is crucial. However, maximal activation of muscle strength may be constrained by various factors, including perceived ability or effort to shift activation towards lower extremity muscles. Monteiro et al. [14] argued that older individuals with higher muscle strength tended to have superior dynamic and static balance, resulting in a reduced perceived risk of falling. Addressing balance impairments is a key objective of well-designed physical exercise programs tailored for older adults. The findings of this study suggest that in preventive diagnosis of physical fitness decline, assessing dynamic balance, along with lower body muscle strength and BMI, should be taken into consideration. Enhancing dynamic balance through improvements in its determinant factors, such as lower limb strength and BMI, may have a positive impact on fall prevention. Given that aging is associated with a decline in muscle mass and a potential increase in body fat, older individuals are prone to exhibit lower levels of physical capacity [28]. Evidence indicates that the decrease in muscle strength, endurance, and mass may be linked to diminished motor functionality and control [14,29], which in turn leads to a decline in dynamic balance. Thus, there is some evidence suggesting that a lower capacity to maintain static or dynamic balance is associated with an increased risk of falls [14]. However, consensus is lacking in the literature, and further research is warranted to explore the influence of muscle mass and muscle strength on static and dynamic balance.

Our findings corroborate previous research [4,30] that has highlighted a strong association between greater muscle strength, as measured by the chair-stand test, and dynamic balance, as assessed by the Timed-Up and Go test. A prior study [14] demonstrated that lower limb muscle strength serves as a predictor for various gait parameters, thus supporting the validity of the proposed model in this investigation. However, it is important to note that the chair-stand test encompasses multiple motor capacities related to muscle strength, including strength, endurance, coordination, and balance [14]. Future studies should incorporate measures of muscle mass to examine the combined association of muscle mass and muscle strength with dynamic balance in older adults.

The current study suggests several areas for intervention to enhance or preserve physical fitness in the later stages of life. Firstly, increasing physical strength in the elderly population may lead to improved balance scores, subsequently reducing the risk of falling. For instance, Forte et al. [31] observed that enhanced physical fitness components, such as muscular activation and balance, were associated with improved overall health status in older individuals. Monteiro et al. [11] implemented a multicomponent training program aimed at improving physical fitness and decreasing body mass index in the elderly, providing evidence for a significant association between increased muscle strength and a reduction in body fat ratio. Thus, increasing muscle mass is advantageous as it is linked to improved physical fitness. However, it should be noted that decreasing body mass index may not always correspond to an increase in physical fitness. In cases of sarcopenia and cachexia, a low body mass index may indicate muscle loss rather than low body fat. In such conditions, enhancing muscle mass and strength becomes crucial for enhancing functional outcomes and quality of life. Therefore, increasing body mass index through the augmentation of muscle mass would likely benefit individuals with sarcopenia or cachexia. Secondly, considering the strong relationship between aging and body composition, engaging in regular physical activity may be particularly beneficial in preventing age-related health decline. Physical activity can help counteract many of the changes associated with aging by promoting muscle activation and increasing metabolic rate. Lastly, a deeper understanding of the physiological and psychological determinants of dynamic balance, as well as factors related to fall risk exposure, would facilitate the development of effective fall prevention programs.

Regarding the relationship between gait speed and falls, previous studies have indicated that gait parameters beyond speed may play a crucial role [32,33]. It has been suggested that measures of gait variability, which reflect the control processes of the brain, could be more strongly associated with fall incidence than gait speed alone [33]. Therefore, future investigations should consider incorporating measures of gait variability to gain a more comprehensive understanding of the relationship between gait and falls in older adults. In our study, we found a significant association between lower body muscle strength and gait speed, suggesting that greater muscle strength may contribute to improved gait performance. However, it is important to note that our model accounted for only a portion of the variance in gait, leaving room for other factors to explain the remaining variability. These factors might include other aspects of physical fitness, such as muscle power or flexibility, which were not assessed in our study. Additionally, cognitive function, sensory impairments, and psychological factors may also influence gait and contribute to the unexplained variance. It is crucial to acknowledge the complexity of gait and falls in older adults and the need for a comprehensive understanding of the underlying mechanisms. While our study contributes to the existing literature by highlighting the association between lower body muscle strength, gait speed, and falls, further research should explore additional variables that could potentially explain the remaining variance. By incorporating a broader range of physiological, cognitive, and psychosocial factors, we can enhance our understanding of the multifactorial nature of gait-related fall risks in older adults [34]. Overall, this study supports the notion that gait speed and lower body muscle strength play important roles in gait performance and fall prevention. However, the current model’s ability to explain only 50% of the variance in gait highlights the need for future research to investigate other factors that may contribute to the unexplained variability. By considering measures of gait variability and exploring a wider range of potential determinants, researchers can develop more comprehensive models that enhance our understanding of gait-related falls and inform effective interventions for older adults.

### Limitations, Strength and Agenda for Future Research

The present investigation is not without limitations. Firstly, although statistically significant, the sample size in our study was small, which restricts the generalizability of our findings to other populations with diverse characteristics. Therefore, it is recommended that future studies replicate our research with larger and more diverse samples. Additionally, we suggest that future investigations explore potential sex differences in the associations we have identified. While our current findings provide valuable insights into the factors influencing the outcome of interest, it is possible that there are sex-specific effects that were not fully captured in our analyses. Another limitation is the lack of information regarding underlying diseases among the participants, which could have influenced agility and muscle strength. Additionally, we did not quantify the occurrence of previous falls in individuals. Furthermore, follow-up studies could advance our understanding by examining the variables underlying the improvement in lower limb strength through the measurement of morphological changes in muscle mass and neuromuscular adaptations. While our findings offer valuable insights into the relationship between anthropometric measures, muscle resistance, and balance, it is important to acknowledge that our study encompassed a broad age range of older adults. This may limit the generalizability of our results to specific age groups. To address this limitation, future studies could focus on comparing the current results across different age cohorts. This would provide a more nuanced understanding of how the variables of interest may vary throughout the lifespan, particularly in older age groups where age-related factors may exert a greater influence.

One strength of our research lies in the inclusion of physically active, older adults who engaged in exercise programs specifically designed to promote adequate levels of physical fitness. Our results support the significant association between increased strength and decreased body mass index, which may contribute to a reduced risk of falls in physically active, older adults. It is worth noting that dynamic balance was assessed through functional field tests, and thus, future studies should incorporate specific tests to measure and assess fall risk and agility.

## 5. Conclusions

The study findings suggest a potential association between muscle strength and dynamic balance in older adults. The results indicate that greater muscle strength may be related to improved dynamic balance in community-dwelling, older individuals. Additionally, lower body mass index scores appear to be associated with better dynamic balance. However, it is important to note that the cross-sectional nature of the study design limits our ability to establish a causal relationship between muscle strength, balance, and functional capacity in the elderly.

While muscle strength is considered an important component of physical fitness, it is crucial to recognize that achieving acceptable levels of agility and balance is essential for the effective performance of daily tasks in older adults. Therefore, interventions such as strength and balance training may have potential significance in improving the autonomous walking ability and enhancing functional capacity in this population. However, it is important to exercise caution in interpreting these findings, as they are based on observations and do not provide definitive evidence of a causal effect. Further research utilizing longitudinal designs and rigorous methodologies is needed to establish a clearer understanding of the relationship between muscle strength, balance, and functional capacity in older adults.

## Figures and Tables

**Table 1:** Descriptive statistics and correlations.

Variables	Units	M	SD	S	K	Correlation Matrix			
						1	2	3	4
1. Age	years	70.31	9.90	0.28	−0.34	1			
2. Body Mass Index	kg/m^2^	27.40	3.62	0.87	0.49	0.36 **	1		
3. Chair-Stand	repetitions	19.96	6.85	0.32	−0.64	−0.35 **	−0.20 *	1	
4. Timed-Up-Go Test	seconds	6.35	4.52	2.20	1.30	0.64 **	0.48 *	−0.38 **	1

**Notes:** M = Mean; SD = Standard-Deviation; S = Skewness; K = Kurtosis; * *p* < 0.05; ** *p* < 0.01.

**Table 2:** Standardized beta coefficients and explained variance.

Model	β	SE	t	R	R_a_^2^	Δ*R*^2^	*p*-Value
Block 1 (B1)				0.382	0.136	0.136	<0.001
Lower body muscle strength	−0.38 **	0.07	−3.77				
Block 2 (B2)				0.567	0.321	0.175	<0.001
Lower body muscle strength	−0.30 **	0.06	−3.19				
Body Mass Index	−0.43 **	0.11	−4.60				
Block 3 (B3)				0.713	0.509	0.188	<0.001
Lower body muscle strength	−0.16 *	0.06	−1.85				
Body Mass Index	0.28 **	0.11	3.32				
Age	−0.49 **	0.04	5.56				

**Notes:** β = standardized coefficients; SE = Standard Errors; t = *t*-test; R_a_^2^ = adjusted r square; Δ = differences. * *p* < 0.05; ** *p* < 0.01

## Data Availability

Data is available under request to the first author.

## References

[1] Marques D.L., Neiva H.P., Marinho D.A., Marques M.C. (2022). Manipulating the Resistance Training Volume in Middle-Aged and Older Adults: A Systematic Review with Meta-Analysis of the Effects on Muscle Strength and Size, Muscle Quality, and Functional Capacity. Sport. Med..

[2] Milanović Z., Pantelić S., Trajković N., Sporiš G., Kostić R., James N. (2013). Age-Related Decrease in Physical Activity and Functional Fitness among Elderly Men and Women. Clin. Interv. Aging.

[3] American College of Sports Medicine (2021). ACSM’s Guidelines for Exercise Testing and Prescription.

[4] Rodrigues F., Domingos C., Monteiro D., Morouço P. (2022). A Review on Aging, Sarcopenia, Falls, and Resistance Training in Community-Dwelling Older Adults. Int. J. Environ. Res. Public Health.

[5] Wilkinson D.J., Piasecki M., Atherton P.J. (2018). The Age-Related Loss of Skeletal Muscle Mass and Function: Measurement and Physiology of Muscle Fibre Atrophy and Muscle Fibre Loss in Humans. Ageing Res. Rev..

[6] Caspersen C.J., Powell K.E., Christenson G.M. (1985). Physical Activity, Exercise, and Physical Fitness: Definitions and Distinctions for Health-Related Research. Public Health Rep..

[7] Rodrigues F., Monteiro A.M., Forte P., Morouço P. (2023). Effects of Muscle Strength, Agility, and Fear of Falling on Risk of Falling in Older Adults. Int. J. Environ. Res. Public Health.

[8] Kalyani R.R., Corriere M., Ferrucci L. (2014). Age-Related and Disease-Related Muscle Loss: The Effect of Diabetes, Obesity, and Other Diseases. Lancet Diabetes Endocrinol..

[9] Hashim N.N.A., Mat S., Myint P.K., Kioh S.H., Delibegovic M., Chin A.-V., Kamaruzzaman S.B., Hairi N.N., Khoo S., Tan M.P. (2023). Increased Body Mass Index Is Associated with Sarcopenia and Related Outcomes. Eur. J. Clin. Investig..

[10] Yoo M.C., Won C.W., Soh Y. (2022). Association of High Body Mass Index, Waist Circumference, and Body Fat Percentage with Sarcopenia in Older Women. BMC Geriatr..

[11] Monteiro A.M., Silva P., Forte P., Carvalho J. (2019). The Effects of Daily Physical Activity on Functional Fitness, Isokinetic Strength and Body Composition in Elderly Community-Dwelling Women. J. Hum. Sport Exerc..

[12] Monteiro A.M., Bartolomeu R.F., Forte P., Carvalho J. (2019). The Effects of Three Different Types of Training in Functional Fitness and Body Composition in Older Women. J. Sport Health Res..

[13] Franco J.R., Jacobs K., Inzerillo C., Kluzik J. (2012). The Effect of the Nintendo Wii Fit and Exercise in Improving Balance and Quality of Life in Community Dwelling Elders. Technol. Health Care.

[14] Monteiro A.M., Forte P., Carvalho J., Barbosa T.M., Morais J.E. (2021). Relationship between Fear of Falling and Balance Factors in Healthy Elderly Women: A Confirmatory Analysis. J. Women Aging.

[15] Chamroonkiadtikun P., Ananchaisarp T., Wajancomkul P. (2021). The Prevalence and Associated Factors of the Fear of Falling in Elderly Patients at the Primary Care Clinic of Songklanagarind Hospital. Top. Geriatr. Rehabil..

[16] Rosário J.L.P. (2014). do Biomechanical Assessment of Human Posture: A Literature Review. J. Bodyw. Mov. Ther..

[17] Singla D., Veqar Z., Hussain M.E. (2017). Photogrammetric Assessment of Upper Body Posture Using Postural Angles: A Literature Review. J. Chiropr. Med..

[18] Herman T., Giladi N., Hausdorff J.M. (2011). Properties of the ‘Timed Up and Go’ Test: More than Meets the Eye. Gerontology.

[19] Soper D. (2022). Factorial Calculator [Software]. https://www.danielsoper.com/statcalc.

[20] Rikli R.E., Jones C.J. (2013). Development and Validation of Criterion-Referenced Clinically Relevant Fitness Standards for Maintaining Physical Independence in Later Years. Gerontologist.

[21] Rodrigues F., Amaro N., Matos R., Mendes D., Monteiro D., Morouço P. (2022). The Impact of an Exercise Intervention Using Low-Cost Equipment on Functional Fitness in the Community-Dwelling Older Adults: A Pilot Study. Front. Physiol..

[22] Tinetti M.E., Richman D., Powell L. (1990). Falls Efficacy as a Measure of Fear of Falling. J. Gerontol..

[23] Cohen J. (1988). Statistical Power Analysis for the Behavioral Sciences.

[24] Durbin J., Watson G.S. (1951). Testing for Serial Correlation in Least Squares Regression. II. Biometrika.

[25] Shumway-Cook A., Baldwin M., Polissar N.L., Gruber W. (1997). Predicting the Probability for Falls in Community-Dwelling Older Adults. Phys. Ther..

[26] Langhammer B., Bergland A., Rydwik E. (2018). The Importance of Physical Activity Exercise among Older People. BioMed Res. Int..

[27] Lin Y.-H., Chen Y.-C., Tseng Y.-C., Tsai S.-T., Tseng Y.-H. (2020). Physical Activity and Successful Aging among Middle-Aged and Older Adults: A Systematic Review and Meta-Analysis of Cohort Studies. Aging (Albany N. Y.).

[28] Sitthiracha P., Eungpinichpong W., Chatchawan U. (2021). Effect of Progressive Step Marching Exercise on Balance Ability in the Elderly: A Cluster Randomized Clinical Trial. Int. J. Environ. Res. Public Health.

[29] Pijnappels M., Reeves N.D., Maganaris C.N., van Dieën J.H. (2008). Tripping without Falling; Lower Limb Strength, a Limitation for Balance Recovery and a Target for Training in the Elderly. J. Electromyogr. Kinesiol..

[30] Shimada H., Tiedemann A., Lord S.R., Suzukawa M., Makizako H., Kobayashi K., Suzuki T. (2011). Physical Factors Underlying the Association between Lower Walking Performance and Falls in Older People: A Structural Equation Model. Arch. Gerontol. Geriatr..

[31] Forte P., Pinto P., Barbosa T.M., Morais J.E., Monteiro A.M. (2021). The Effect of a Six Months Multicomponent Training in Elderly’s Body Composition and Functional Fitness—A before-after Analysis. Motricidade.

[32] Sherrington C., Fairhall N.J., Wallbank G.K., Tiedemann A., Michaleff Z.A., Howard K., Clemson L., Hopewell S., Lamb S.E. (2019). Exercise for preventing falls in older people living in the community. Cochrane Database Syst. Rev..

[33] Verghese J., Holtzer R., Lipton R.B., Wang C. (2009). Quantitative gait markers and incident fall risk in older adults. J. Gerontol. A Biol. Sci. Med. Sci..

[34] Stotz A., Hamacher D., Zech A. (2023). Relationship between Muscle Strength and Gait Parameters in Healthy Older Women and Men. Int. J. Environ. Res. Public Health.

